# LINC00152 promotes proliferation in hepatocellular carcinoma by targeting EpCAM via the mTOR signaling pathway

**DOI:** 10.18632/oncotarget.5970

**Published:** 2015-10-26

**Authors:** Jie Ji, Junwei Tang, Lei Deng, Yu Xie, Runqiu Jiang, Guoqiang Li, Beicheng Sun

**Affiliations:** ^1^ Liver Transplantation Center of the First Affiliated Hospital and Collaborative Innovation Center For Cancer Personalized Medicine, Nanjing Medical University, Nanjing, Jiangsu Province, P.R. China

**Keywords:** lncRNA, HCC, EpCAM, mTOR, prognosis

## Abstract

Hepatocellular carcinoma (HCC) is well known as the sixth most common malignant tumor and the third leading cause of cancer-related deaths globally. LINC00152 was documented as an important long non-coding RNA (lncRNA) involved in the pathogenesis of gastric cancer; however, the detailed mechanism of action of LINC00152 remains unknown. Here, based on the increased level of LINC00152 in HCC tissues, we found that LINC00152 could promote cell proliferation *in vitro* and tumor growth *in vivo*. Furthermore, microarray-based analysis indicated that LINC00152 could activate the mechanistic target of rapamycin(mTOR) pathway by binding to the promoter of EpCAM through a *cis*-regulation, as confirmed by Gal4-λN/BoxB reporter system. Thus, LINC00152 might be involved in the oncogenesis of HCC by activating the mTOR signaling pathway and might be a novel index for clinical diagnosis in the future.

## INTRODUCTION

Every year, 500,000 people are affected by HCC, which is the sixth most common malignant tumor and the third leading cause of cancer-related deaths globally [1, 2]. HCC contains a number of characteristics, including aggressiveness, invasiveness, especially intrahepatically, and frequent recurrence after resection [3]. Despite recent improvements in surgery and chemotherapy, HCC has a very high morbidity and mortality [4]. Therefore, the potential mechanisms, prognostic biomarkers and therapeutic targets of HCC require further investigation [1, 5, 6].

The transcripts of greater than 200 nucleotides with no or little protein coding function, i.e. long non-coding RNAs, has attracted much attention in many fields [7, 8]. Several lncRNAs were reported as biomarkers for predicting metastasis and diagnosis of multiple diseases, including hepatosis and HCC, as well as patient survival [9–12]. Recent studies have identified multiple functional effects of lncRNAs, including regulating gene expression through modulation of chromatin remodeling, controlling gene transcription, post-transcriptional mRNA processing, protein function or localization, and intercellular signaling [13–16]. The mechanisms of lncRNAs involved in liver disease have widely diverse functions, including DNA imprinting, X inactivation, DNA demethylation, gene transcription, and generation of other RNA molecules [17, 18]. Furthermore, several researchers have discovered that lncRNAs could be modified epigenetically including by methylation, ubiquitination, and miRNA-induced regulation through a network [19].

The long noncoding RNA, LINC00152, was detected as differentially hypomethylated during hepatocarcinogenesis compared to healthy samples [20]. LINC00152 was reported to play an important role in cancer and has a 1.9-fold change of expression in gastric cancer [21]. The expression level of LINC00152 was increased significantly in both gastric carcinoma and gastric juice [22]. Meanwhile, LINC00152 can be detected in plasma, and one of the possible mechanisms of its stable existence in blood is that it is protected by exosomes [23]. LINC00152 appears to respond generally and highly to chemical stresses [24]. Besides, researcher has found LINC00152 may interact with THBS1 mediated by miR-18a-5p [25].

In this study, we explored whether LINC00152 is involved in hepatocarcinogenesis. Additionally, we attempted to identify the detailed effects and mechanisms of LINC00152 in HCC, both *in vivo* and *in vitro*.

## RESULTS

### Hypomethylated LINC00152 was up-regulated in the primary HCC tissues and human HCC cell lines

**Figure 1:**
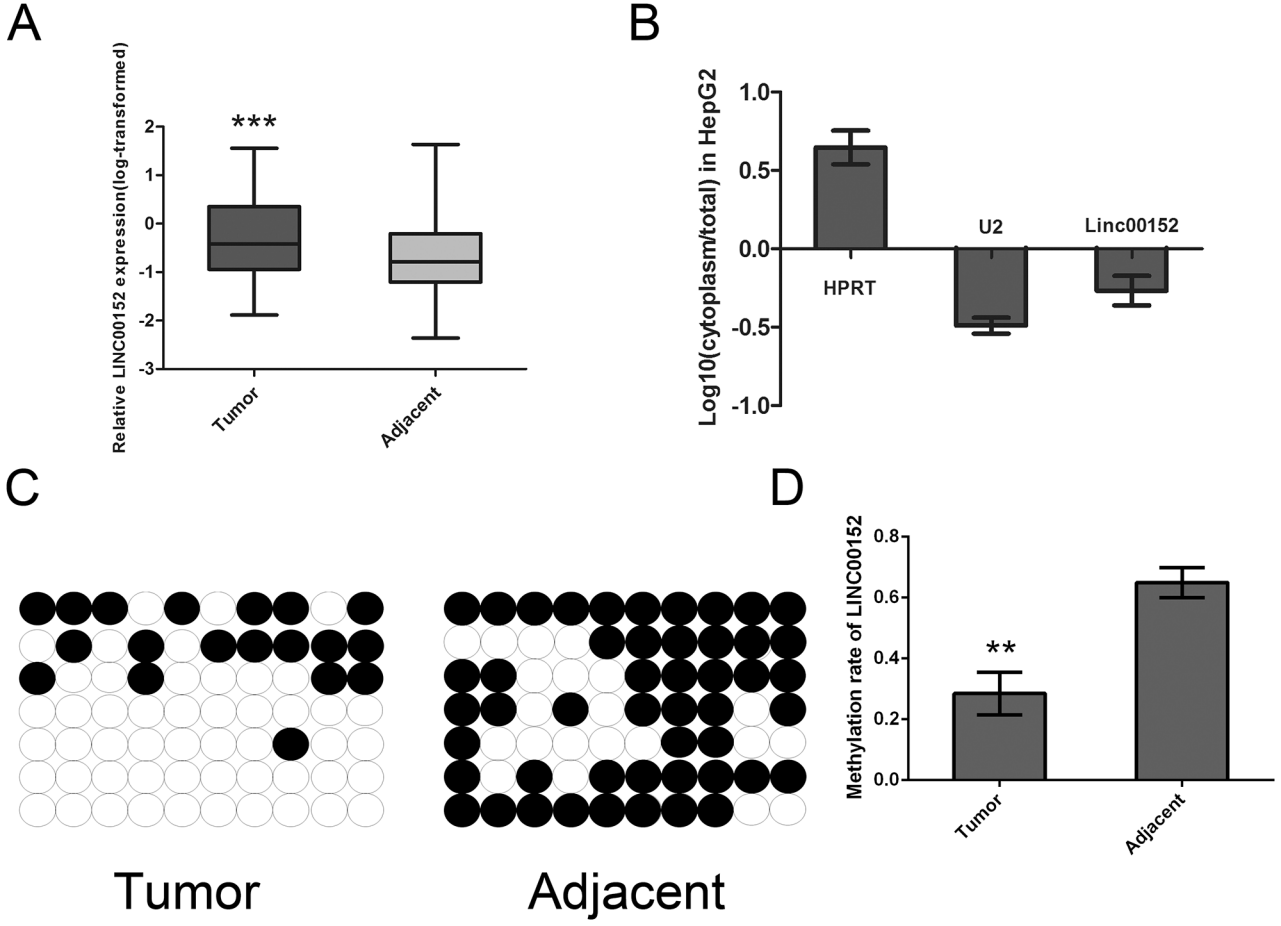
Aberrant up-regulated LINC00152 in HCC tissue samples and the subcellular location **A.** An increased level of LINC00152 was detected in HCC tissues compared with the corresponding adjacent tissues (*n* = 102). All the expression of the LINC00152 normalized to 18S using the 2^−ΔΔCt^ method, then the expression of LINC00152 was transformed with log_10_. **B.** Subcellular localization investigation indicated that the transcript for LINC00152 was located mainly in the nucleus of MHCC-97H cell lines, according to the results of RT-PCR amplification with separated cytoplasmic RNA and nuclear RNA. HPRT was used as the control for cytoplasmic expression and U2 for cytonuclear expression. **C, D.** The bisulfite sequencing (BSP) method was used to detect methylation of CpG Island predicted in LINC00152. The methylation level of the LINC00152 promoter was downregulated in tumor tissues compared with the corresponding adjacent tissues. (** indicates *p* < 0.01 while *** indicates *p* < 0.001).

To investigate the LINC00152 expression level in HCC, we performed RT- PCR analysis on total RNA extracted from 102 pairs of HCC tissues. The expression level of LINC00152 in hepatocellular carcinoma was significantly increased, compared with matched normal tissue (Figure 1A). In addition, all HCC samples were divided into the LINC00152 low expression group (*n* = 51) and the high-expression group (*n* = 51), and the median was used as the cut-off value. The association between LINC00152 expression in HCC tissues and clinicopathological characteristics is presented in Table 1. We found no positive correlation with age, gender, HBV infection, cirrhosis, ALT, AFP, and vascular invasion. However, significant correlations with tumor size (*P* = 0.005) and Edmondson grade (*P* = 0.002) were obtained, indicating that LINC00152 might play a vital role in the development of HCC.

**Table 1:** Correlation between LINC00152 expression and clinicopathological characteristics of HCC patients (*n* = 102)

Characteristics	All Patients	LINC00152 Low Expression(< Median^a^)	LINC00152 High Expression(≥ Median^a^)	pChi-squared test *p*-value
**No.**	102	51	51	
**Age (years)**				
< 60	77	35	42	0.107
≥ 60	25	16	9	
**Gender**				
Male	89	45	44	0.862
Female	13	6	7	
**HbeAg**				
Negative	29	14	15	0.826
Positive	73	37	36	
**Cirrhosis**				
Absent	16	7	9	0.586
Present	86	44	42	
**ALT (U/L)**				
≤ 45	59	29	30	0.841
>45	43	22	21	
**AFP (ng/ml)**				
≤ 13.6	23	10	13	0.477
>13.6	79	41	38	
**Tumor size (cm)**				
≤ 5	42	28	14	0.005*
>5	60	23	37	
**Vascular invasion**				
absent	87	45	42	0.402
present	15	6	9	
**Edmondson grade**				
I+II	61	38	23	0.002*
III+IV	41	13	28	

a The median expression level of Linc-152 was used as the cutoff

* Indicates *p* value < 0.05

To investigate the subcellular location of LINC00152, the SurePrep™ Nuclear/Cytoplasmic RNA Purification Kit was used. The transcript for LINC00152 was located mainly in the nucleus of MHCC-97H cells (Figure 1B). Further methylation analysis was performed by Bisulfite sequencing PCR (BSP). We found that the promoter region of LINC00152 was hypomethylated in tumor tissues (Figure 1C, 1D). Since LINC00152 has not been reported in human HCC, we then started to analyze the second structure, non-coding function using bioinformatics software. As presented in Supplementary Figure S1A–S1C, LINC00152 was confirmed as the long non-coding RNA with a PhyloSCF score of −75.9943.

### LINC00152 promotes cell proliferation *in vitro*

To determine the functional role of LINC00152 in HCC, we first detected the expression of LINC00152 in the following six human HCC cell lines: HepG2, MHCC-97H, Huh7, SMMC-7721, Hep3B and SNU-423. As shown in Supplementary Figure S1D, LINC00152 expression levels were up-regulated in HCC cell lines compared to that in L02 normal human liver cells. Therefore, HepG2 and MHCC-97H were selected to investigate the effects of shRNA-mediated knockdown of LINC00152 on cell proliferation, apoptosis, invasion, migration and cell cycle.

Three LINC00152-specific shRNAs were evaluated for their knockdown efficiency, sh152-1 and sh152-3 were found to have higher silencing efficiency than sh152-2 (Supplementary Figure S1E). Thus, sh152-1 and sh152-3 were chosen for use in cell physiological function assays.

**Figure 2:**
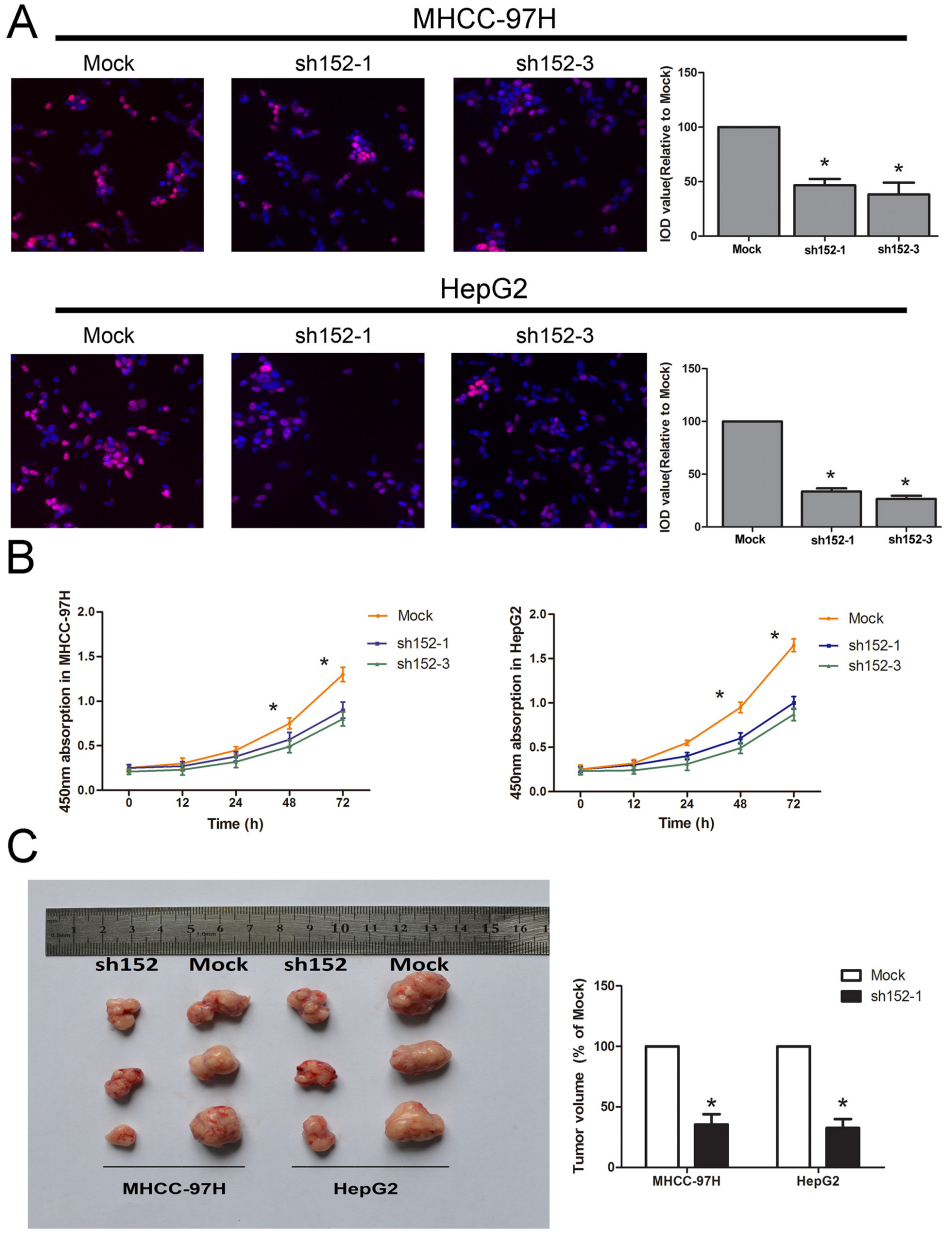
LINC00152 promoted cell proliferation *in vitro* and tumor growth *in vivo* **A.** The EdU assay confirmed the functional role of LINC00152 in cell proliferation. Stable knockdown of LINC00152 reduced the proliferation of MHCC-97H and HepG2 after 24 h (*P* < 0.01) compared with the negative control, with a magnification of 200. The integral optical density value of cells treated with control plasmids was normalized to 100%. **B.** The CCK-8 assay presented showed that a decreased level of LINC00152 inhibited the growth of MHCC-97H and HepG2. Absorbance at 450 nm is presented as the mean ± SEM. **C.** All experiments were performed in triplicate and presented as the mean ± SEM. * indicates a significant difference compared with the control group (*P* < 0.05). C: Bilateral axillae of BALB/C nude mice were transplanted subcutaneously with MHCC-97H or HepG2 cells stably expressing LINC00152 shRNA or the mock (*n* = 5). The volume of each tumor was calculated as the length × width^2^ × 0.5. The tumor volume of cells treated with controls was normalized to 100%. Data are presented as the mean ± SEM. * indicates a significant difference compared with controls (*P* < 0.05).

The data collected using the Cell Counting Kit-8 (CCK-8) and EdU (5-ethynyl-2′-deoxyuridine) cell proliferation assays suggested that down-regulation of LINC00152 inhibited the proliferation in both HepG2 and MHCC-97H cells (Figure 2A, 2B). Meanwhile, invasiveness, cell cycle and apoptosis analysis showed no significant difference between the sh152-treated cells and the normal control, in both of the cell lines mentioned above (Supplementary Figure S2A–S2C).

### LINC00152 induced a promotion of tumor growth *in vivo*

To confirm the effects of LINC00152 on tumorigenesis *in vivo*, we conducted a nude mice xenograft experiment by subcutaneously injecting HepG2 and MHCC-97H cells whose LINC00152 was stably knocked down, or a mock vector (cells treated with a mock vector) into the flanks of approximately 4–6-week-old BALB/C nude mice. The left axilla was injected with mock cells, while the right side was injected with cells treated with sh152-1. We observed that tumor growth was significantly decreased compared with the controls (Figure 2C).

### The mTOR signal pathway was inhibited by the down-regulation of LINC00152 through a *cis*-regulation

**Figure 3:**
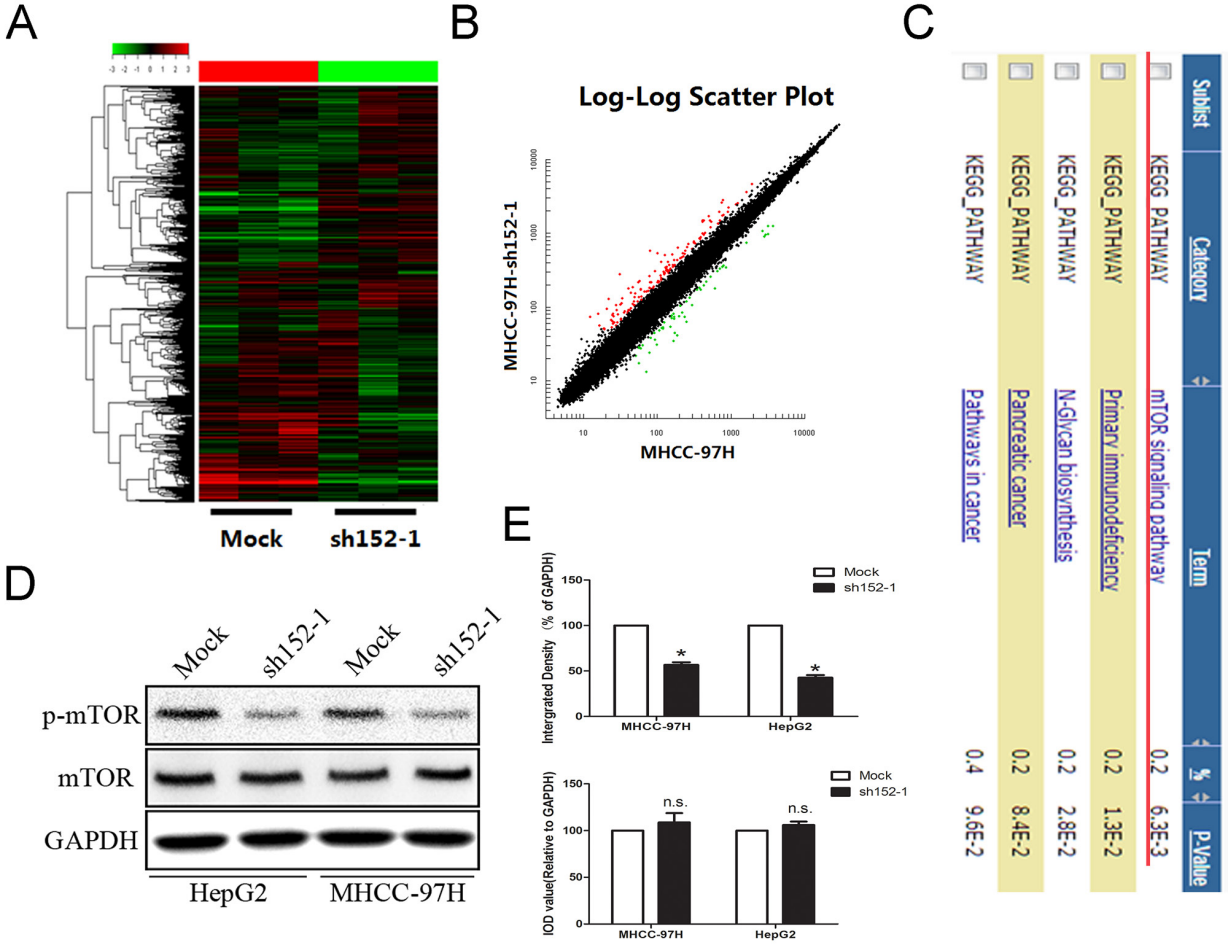
Microarray investigation indicated that the mTOR signal pathway was suppressed by the absence of LINC00152 **A, B.** Heatmap analysis and scatter plot results present different gene expression with the cutoff value set at 4 / 0.25. **C.** Gene annotation for enrichment of the candidate genes. **D.** Western blot was applied to confirm that the aberrant suspension of the mTOR signaling pathway was induced by the loss of LINC00152. GAPDH was used as a loading control. **E.** Integral optical density (IOD) was calculated for each band. Data are presented as the mean ± SEM. * indicates *P* < 0.05.

LINC00152 was reported to be up-regulated in multiple cancers and may play an important role in post-transcriptional regulation in cancer [22]. However, the specific signaling pathway involved in the abnormal expression of LINC00152 remained unknown. A microarray-based study was employed to demonstrate the potential signaling pathways. MHCC-97H cells were divided into two groups, including LINC00152 stable knockdown and the mock plasmid-treated cells. As presented in Figure 3A and 3B, genes with aberrant expression were selected with 4 / 0.25 as the cut-off which were regarded as candidate genes for Gene Set Enrichment Analysis. Gene-annotation enrichment analysis indicated that the mTOR signal pathway was highly associated with LINC00152 down-regulation (Figure 3C). The anomalous activation of genes participating in the signaling pathway was confirmed by Western blot. As a result, the phosphorylation level of mTOR was decreased dramatically, along with the absence of LINC00152 in HepG2 and MHCC-97H cells (Figure 3D, 3E).

**Figure 4:**
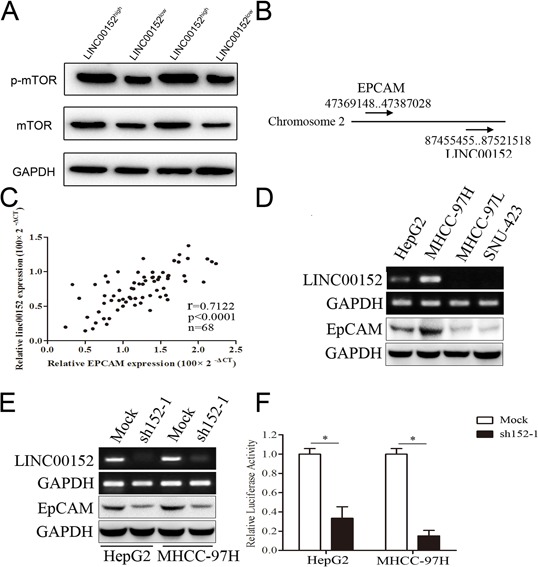
LINC00152 activated mTOR via binding the promoter of EpCAM **A.** Different expression of mTOR and *p*-mTOR in human tissues in LINC00152^low^ and LINC00152^high^ groups. **B.** Location of LINC00152 and EpCAM in Chromosome 2. **C.** Correlation expression levels of LINC00152 and EpCAM using Pearson analysis. **D.** Different RNA expression levels of LINC00152 and EpCAM in cell lines. **E.** RNA expression level of LINC00152 in cells treated with sh152-1 and control (top panel). Protein expression level of EpCAM in cells treated with sh152-1 and control (lower panel). **F.** Cells were co-transfected with the promoter region of EpCAM with reporter, the *Renilla* luciferase vector pRL-SV40 and LINC00152 shRNA, or control for 48 h. Both firefly and *Renilla* luciferase activities were measured in the same sample. Firefly luciferase signals were normalized with *Renilla* luciferase signals. Cells treated with controls of miRNA were normalized to 100%.

The HCC patients were divided into two groups (LINC00152^low^ and LINC00152^high^) according to the median of LINC00152 expression in tumor tissues. We also analyzed the expression of mTOR and p-mTOR in the two groups described above. We found that the phosphorylation level of mTOR was decreased along with the lower expression of LINC00152 (Figure 4A). We next retrieved information from the National Center for Biotechnology Information (NCBI) Gene database (http://www.ncbi.nlm.nih.gov/gene) and found an mTOR-related gene, EpCAM, located near LINC00152 (Figure 4B). EpCAM is expressed in many human cancers with an epithelial origin. EpCAM has been implicated in cell invasion and may act as an oncogenic signaling protein. EpCAM(+) HCC cells displayed hepatic cancer stem cell-like traits, including the abilities to self-renew and differentiate. Moreover, these cells were capable of initiating highly invasive HCC [26–29]. In our study, we further investigated the correlation between LINC00152 and EpCAM in clinical HCC tissues, and found that the expression levels of LINC00152 and EpCAM were positively correlated (Figure 4C). Then, we confirmed that the expression levels of LINC00152 and EpCAM were both decreased in MHCC-97L and SNU-423 cells when compared with HepG2 and MHCC-97H cells, using PCR and Western blot (Figure 4D). Interestingly, we found that silencing LINC00152 by sh152-1 resulted in the down-regulation of EpCAM in HepG2 and MHCC-97H cells at both the mRNA and protein levels (Figure 4E). Then, we cloned the 5(−flanking region of EpCAM (−2000 bp region), regarded as the promoter region, into the pGL3-Basic vector (pGL3-EpCAM). Dual luciferase reporter gene assay showed that the knockdown of LINC00152 could decrease the promoter activity of EpCAM in HepG2 and MHCC-97H cells (Figure 4F).

**Figure 5:**
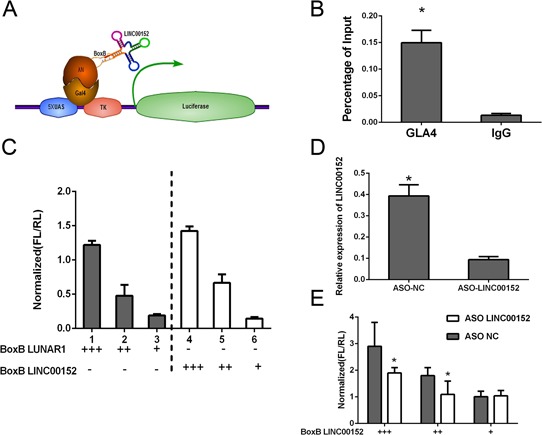
Cis-regulation of LINC00152 **A.** Schematic figure for Gal4-λN/BoxB reporter system. **B.** The binding of Gal4-λN fusion was confirmed by ChIP. **C.** Luciferase reporter activity in experiments where BoxB-tagged LINC00152 (right) or LUNAR1 (left) were cotransfected with Gal4-lN. **D.** qPCR following ASO knockdown of LINC00152 in luciferase assay. **E.** Reporter assay showing relative reporter gene activity when BoxB-LINC00152 was cotransfected with either control or ASO targeting LINC00152. Data was normalized to Ranilla luciferase. Data presented as the mean ± SEM. * indicates *p* value < 0.05.

Gal4-λN/BoxB reporter system was employed as described previously to explore whether LINC00152 regulated EpCAM as a *cis*-pattern [30, 31]. In this system, the BoxB RNA stem loop is fused to LINC00152; LUNAR1 was used as a positive control. The plasmid encoding a TK-luciferase gene under the control of five GAL4 UAS sites was co-transfected with plasmids encoding GAL4-λN peptide as described above (Figure 5A). Ranilla luciferase was regarded as control in this system. The binding of Gal4-λN fusion was confirmed firstly (Figure 5B). Luciferase activity after co-transfection of the system indicated that tethering LINC00152 to this reporter gene could stimulate transcription of the reporter to a similar degree as LUNAR1 indicating that LINC00152 could function as a transcription activator (Figure 5C).

Further antisense oligonucleotides (ASO) targeting LINC00152 was designed, we found that the activation affection was significantly reduced comparing to control group (Figure 5D, 5E).

## DISCUSSION

HCC is a worldwide disease with a high incidence. Despite advanced therapeutic treatments such as surgery, chemotherapy and radiotherapy, more than 600,000 people die of HCC each year [32]. Hence, the exploration of the pathogenesis and development of effective treatments is essential. A number of LncRNAs have been discovered to play a vital role in hepatocarcinogenesis and HCC progression [33], including, HULC [34], HOTTIP [14], HOTAIR [35], MVIH [36]. It has been documented that the expression of LINC00152 in gastric cancer is higher compared to normal gastric tissue [21].

Along the digestive tract, we detected abnormal expression of LINC00152 in HCC, which was significantly correlated with tumor size. We then confirmed the proliferative promotion of LINC00152 *in vitro* and *in vivo*. To further investigate the potential signaling pathway and target gene regulated by LINC00152, we used microarray and bioinformatics analyses. Based on the data of the transcriptome microarray assay, signaling pathway enrichment and target gene differential analysis, we discovered that LINC00152 potentially regulates the mTOR signaling pathway, and confirmed this with Western bolting. We therefore used the dual luciferase reporter gene assay to explore how LINC00152 targets EpCAM at the transcriptional level. We found that the knockdown of LINC00152 could decrease the promoter activity of EpCAM in HepG2 and MHCC-97H cells. Combined with other research, we found that the direct interaction between lncRNAs and the promoter of target genes is one of the major regulatory paradigms of lncRNAs.

Cis-acting lncRNAs whose regulatory functions are restricted to genes on the same chromosome are likely to operate only at the level of transcription [37]. Taro et al. identified two TCF binding elements (TBE1; -TTCAAAG-, TBE2; -CTTTGAT-) in the EpCAM promoter that specifically bind to the TCF/β-catenin complex. We found two sequences (432–438; -CAUUGAA-, 609–616; -GAUCACAG-) in the LINC00152 that may bind to TBE1 and TBE2 [29]. In human distal cells, the lncRNA HOTTIP recruits the histone H3K4-modifying complex MLL1 by binding to WDR5, targeting this complex to the HOXA locus. Thus, chromatin modifications together with higher-order chromosomal looping bring the HOTTIP RNA in close proximity to HOXA genes [38, 39]. In our study, the LINC00152 may be required for maintaining transcriptionally active EpCAM genes at the 5′ end of the cluster.

In conclusion, our findings demonstrate the up-regulation of LINC00152 in human HCC,. Aberrant expression of LINC00152 increases EpCAM levels, resulting in the activation of the mTOR signaling pathway, and afterwards, causing the proliferation of HCC both *in vitro* and *in vivo*. These findings contribute to a better understand of the mechanism of action of lncRNA. Our data indicate that LINC00152 might be involved in HCC development, which may play a vital role in the tumorigenesis of HCC, and may serve as potential target for therapy in the future.

## MATERIALS AND METHODS

### Clinical samples and cell cultures

The clinical data were obtained from 102 cases of patients who underwent liver cancer radical resection surgeryduring January 2010 to December 2012 at The First Affiliated Hospital of Nanjing Medical University (Nanjing, China). The specimens were stored at −80°C immediately after surgery. No patient had a history of exposure to either radiotherapy or chemotherapy before the surgery, and no other co-occurring cancers were diagnosed. This study was approved by the Ethical Committee of the First Affiliated Hospital of Nanjing Medical University, and every patient provided written informed consent.

The human HCC cell lines used in this study were obtained from the American Type Culture Collection (Manassas, VA, USA), and included HepG2, Hep3B and SNU-423 cells. Huh7, L02, SMMC-7721 and MHCC-97H cells were purchased from the Cellbank of the Chinese Academy of Sciences in Shanghai. All of the cell lines were maintained in an atmosphere of 5% CO_2_ and grown in DMEM medium (Thermo, Beijing, China) supplemented with 10% fetal bovine serum (Thermo). Cell line authentication was performed by short tandem repeat (STR) profiling before the initiation of this study.

### Quantitative real time polymerase chain reaction (qRT-PCR)

Quantitative real time polymerase chain reaction (qRT-PCR) was performed to determine the expression levels of LINC00152 and other related mRNAs. Total RNAs were extracted from frozen tissues and cell lines using TRIzol reagent, as described by the manufacturer's protocol (Invitrogen, CA, USA). One microgram of total RNA from all samples was reverse transcribed using the PrimeScript RT Master Mix kit (Takara, Tokyo, Japan). Beta-actin was used as an internal control. All the primer sequences are shown in Supplementary Table S1. qRT-PCR was performed using an ABI Prism 7900HT instrument (Applied Biosystems, CA, USA), according to the instructions of the manufacturer. The gene expression data were analyzed using the 2^−ΔΔCt^ method.

### Gene silencing of LINC00152 in HCC cell lines

To knock down LINC00152 expression, an shRNA sequence targeting LINC00152 was cloned into the lentivirus vector pLL3.7, and designated as pLL3.7-sh152-1 and pLL3.7-sh152-2. The vectors also included the gene for green fluorescent protein (GFP). The sequences are presented in Supplementary Table S1. The hairpin shRNA with no sequence homology to human genes provided by the same manufacturer was used as the negative control, named the mock group. All constructs were sequence-verified. Details are available on request. Recombinant lentivirus was generated from 293T cells by cotransfection of pdelta-8.91, pVSVG together with pLL3.7-sh152-1 and pLL3.7-sh152-2. The GFP-positive cells were sorted 3 days after infection with the lentiviruses. The stable cells infected with LINC00152 shRNA were selected by puromycin. After cells treated with lentivirus for 48 h, medium containing puromycin was added and the medium was replaced every 2 days. A pure population of infected cells was sorted based on GFP-expression by flow cytometry; more than 98% of the cells were GFP-positive after sorting. A total of 1.5 × 10^5^ cells were seeded in 35 mm^2^ tissue culture dishes for 24 h. The knock-down efficiency of the LINC00152 by shRNAs was monitored by qRT-PCR. The cells were then subjected to RNA extraction or functional assays.

### Cell proliferation assay

Cell proliferation was assayed using CCK-8(Dojin Laboratories, Kumamoto, Japan) and EdU(Millipore, MA, America), according to the manufacturer's instructions. The mock and infected cells were seeded at a density of 1 × 10^4^ cells/well in 96-well flat-bottom plates, and cultured for the CCK-8 and EdU assays.

After a period of time (0, 12, 24, 48, 72 h) following the seeding of the cells, CCK-8 was added to each well containing 100 μL of the culture medium, and the plate was incubated for 3 h at 37(C. Viable cells were evaluated by measuring the absorbance at 450 nm, using a microplate reader. The EdU assay was performed after the cells were cultured for 48 h. Finally, the cells were washed, mounted with mounting medium containing DAPI (Santa Cruz Biotechnology, CA, USA), and imaged using an Olympus FV1000 confocal microscope (Olympus, NY, USA).

### Cell cycle and apoptosis analysis

Cells were infected with sh152-1, as well as with normal controls, and were harvested 48 h later. For the cell cycle assay, cells were fixed in 75% alcohol, and stained with propidium iodide (Sigma, MO, USA). For the analysis of apoptosis, cells were washed in PBS, and then mixed with the Annexin V-FITC Apoptosis Detection Kit (BD Biopharmingen, NJ, USA) for 15 min in the dark. All experiments were analyzed using the BD Biosciences FACSCalibur Flow Cytometer (BD Biasciences, NJ, USA). The tests were repeated three times, and triplicate wells were used for each condition.

### The subcutaneous xenotransplantation model

Animal care and euthanasia were approved by the Nanjing Medical University Animal Studies Committee. HepG2 and MHCC-97H cells and control cells (1 × 10^7^) stably knocked down for LINC00152 expression were implanted subcutaneously into the bilateral axillae of twelve BALB/C nude mice. Tumors were measured every week after implantation, and the volume of each tumor was calculated (length × width^2^ × 0.5). All mice were sacrificed 5 weeks afterwards, and the xenografts were peeled off subcutaneously. The weights of the xenografts in each group were compared.

### Protein extraction and Western blotting

Total protein was extracted from tissues or cultured cells using RIPA buffer containing phenylmethanesulfonylfluoride (PMSF) (Beyotime, Nantong, China). Protein samples were loaded equally in each lane, then resolved using SDS–PAGE (Beyotime, Nantong, China), and transferred onto a nitrocellulose membrane. The membranes were blocked with 5% BSA for 1 h at room temperature and incubated at 4°C overnight with primary antibodies purchased from Abcam (London, UK). Sequentially, the secondary antibodies were conjugated to horseradish peroxidase, and the proteins were visualized via chemiluminescence (Millipore, CA, USA). GAPDH (Abcam, London, UK) was used to normalize the quantity of the protein. The integrated density of the band was quantified using Image Lab software (Bio-Rad, CA, USA).

### Microarray assay and bioinformatics analyses

Total RNA was extracted from HepG2 and MHCC-97H cells in which LINC00152 was stably knocked down, and control cells were treated with the corresponding empty plasmid, PLL3.7, and was amplified and transcribed into fluorescent cDNA. Labeled samples were hybridized to the Human Roche NimbleGen mRNA microarray (Roche, CA, USA). Bioinformatics analyses were conducted using the MAS3.0 system (CapitalBio, Beijing, China) and DAVID Functional Annotation Bioinformatics Microarray Analysis (http://david.abcc.ncifcrf.gov/).

### Gal4-λN/BoxB reporter assay

In this system, the BoxB RNA stem loop is fused to LINC00152, LUNAR1 was used as a positive control as described previously [30]. The plasmid encoding a TK-luciferase gene under the control of five GAL4 UAS sites was co-transfected with plasmids encoding GAL4-λN peptide fused to a C-terminal GFP tag, BoxB as described above. Ranilla luciferase was regarded as control in this system. The binding of Gal4-λN fusion was confirmed firstly.

### ASO technology

Antisense oligonucleotides (ASOs) were designed using the IDT Antisense Design Tool (http://www.idtdna.com) using the chimeric 25-mer setting. The top 3 ASOs generated by the design tool were ordered and tested for knockdown efficiency for further investigation. Sequence for the control group in ASO assay was taken from Thomas Trimarchi, et al [30]. For ASO knockdown in BoxB tethering experiments, ASOs were co-transfected with plasmid DNA at 50 nM.

### Dual luciferase reporter gene assay

The EpCAM promoter sequence (−2000 bp) was cloned into the plasmid pGL3-Basic. The treated cells harvested 48 h after siRNA treatment, and the firefly luciferase expression was measured and normalized to *Renilla* activities. Dual-luciferase assays (Promega, Madison, WI) were performed according to the manufacturer's protocol, and detected with a Fluoroskan microplate reader (Thermo Labsystems, Helsinki, Finland).

### Statistical analysis

All experiments were performed in triplicate, and repeated at least three times. Data were expressed as mean ± SD. Differences between two independent groups were tested with Student's *t*-test. All statistical analyses were carried out using SPSS version 18.0 and presented with Graphpad prism software. The results were considered to be statistically significant at *P* < 0.05.

## SUPPLEMENTARY FIGURES AND TABLE


