# Supplementary material for: LINC00152 promotes proliferation in hepatocellular carcinoma by targeting EpCAM via the mTOR signaling pathway
Source: Oncotarget. 2015 Oct 26;6(40):42813–24. doi: 10.18632/oncotarget.5970 (PMC4767473; doi:10.18632/oncotarget.5970)
Supplement: Supplementary file 1 [file oncotarget-06-42813-s001.pdf]

SUPPLEMENTARY FIGURES AND TABLE

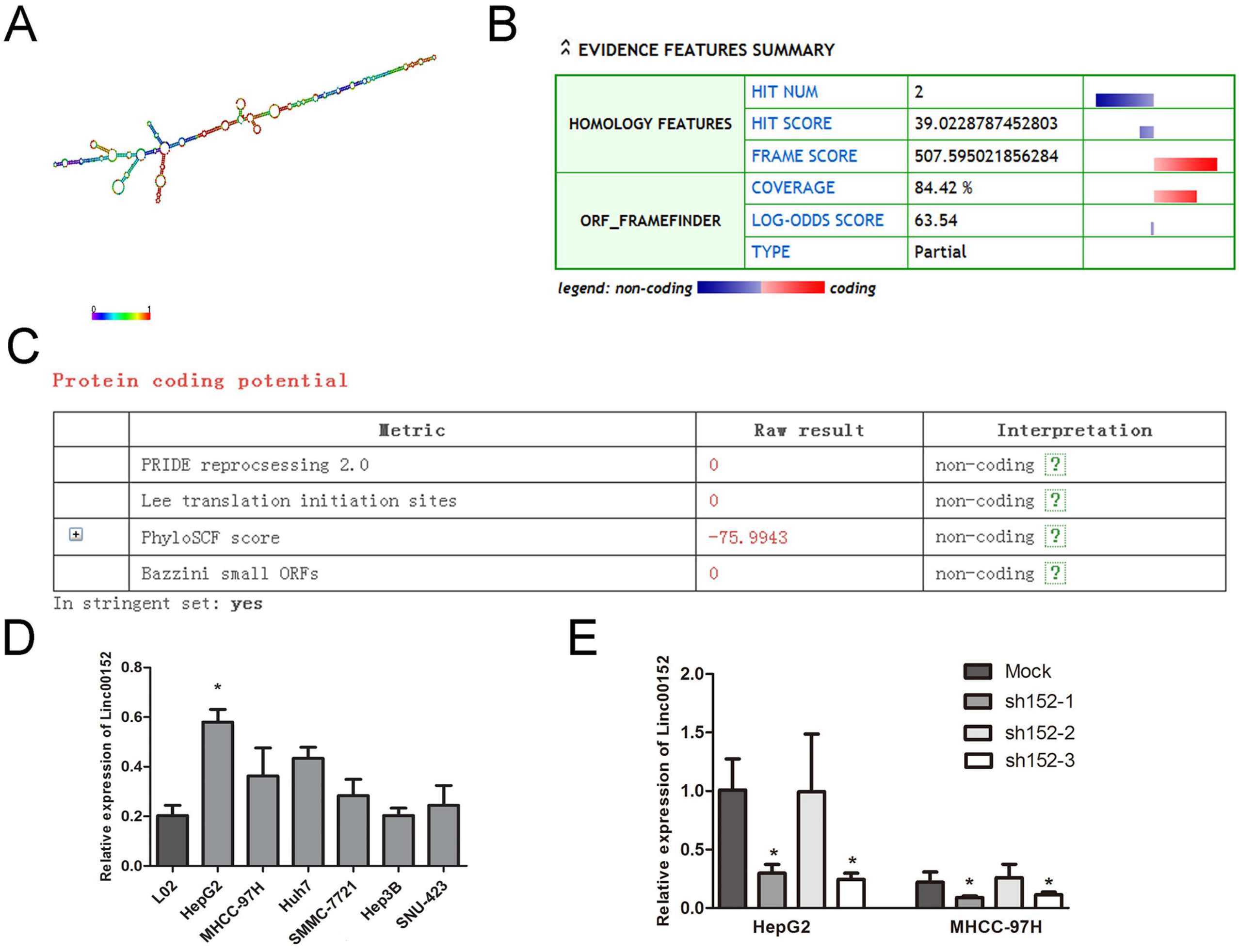

EVIDENCE FEATURES SUMMARY

HOMOLOGY FEATURES	HIT NUM	2	
	HIT SCORE	39.0228787452803	
	FRAME SCORE	507.595021856284	
ORF_FRAMEFINDER	COVERAGE	84.42 %	
	LOG-ODDS SCORE	63.54	
	TYPE	Partial	

legend: non-coding coding

Protein coding potential

	Metric	Raw result	Interpretation
	PRIDE reprocsessing 2.0	0	non-coding ?
	Lee translation initiation sites	0	non-coding ?
+	PhyloSCF score	-75.9943	non-coding ?
	Bazzini small ORFs	0	non-coding ?

In stringent set: **yes**

Supplementary Figure S1: Non-coding RNA validation and the expression of LINC00152 in cell lines. A. The second structure of LINC00152. **B, C.** Non-coding RNA validation by bioinformatics prediction. **D.** Different expression level of LINC00152 in HCC cells. **E.** Expression of LINC00152 in cells treated with shRNA-1, shRNA-2, shRNA-3 and the empty plasmid. Data were presented as the mean ± SEM. * indicates a significant difference compared with controls ($P < 0.05$).

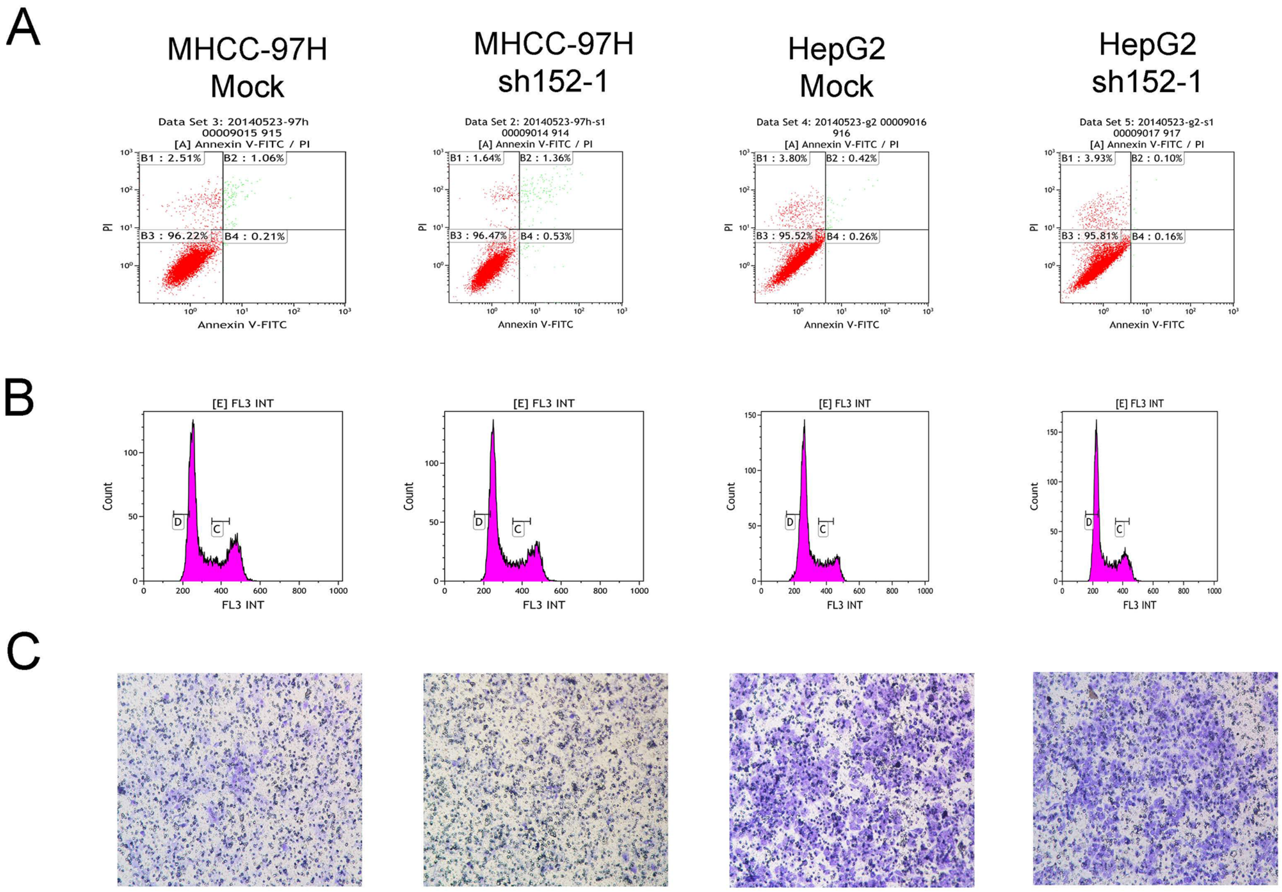

Supplementary Figure S2: No significant of LINC00152 on cell apoptosis, cell cycle or invasion. Cells were treated with shRNA of LINC00152 and empty plasmid in MHCC-97H and HepG2. **A, B.** Apoptosis and cell cycle detection by Flow Cytometry assay. **C.** Invasion was detected by Transwell assay with matrigel.

Supplementary Table S1: Detailed sequence information of primers and shRNAs

	Forward primer (5′-3′)	Reverse primer (5′-3′)
Linc00152	CTCCAGCACCTCTACCTGTTG	GGACAAGGGATTAAGACACACA
EpCAM	GGCTCTTTAAGGCCAAGCAG	CACTCGCTCAGAGCAGGTTAT
GAPDH	GCACCGTCAAGGCTGAGAAC	GGATCTCGCTCCTGGAAGATG
5S RNA	GGAGAGGGAGCCTGAGAAACG	TTACAGGGCCTCGAAAGAGTCC
sh152-1	TCTATGTGTCTTAATCCCTTGTCCT	
sh152-3	TGTGGACTCTGAGGCCTCTGCATTT	
